# Supplementary material for: An artificial protein modulator reprogramming neuronal protein functions
Source: Nat Commun. 2024 Mar 6;15:2039. doi: 10.1038/s41467-024-46308-6 (PMC10917760; doi:10.1038/s41467-024-46308-6)
Supplement: Supplementary file 1 — Supplementary Information [file 41467_2024_46308_MOESM1_ESM.pdf]

An artificial protein modulator reprogramming neuronal protein functions

Peihua Lin[1,2,9], Bo Zhang[1,3,9], Hongli Yang[2], Shengfei Yang[2], Pengpeng Xue[2], Ying Chen[2], Shiyi Yu[2], Jichao Zhang[4], Yixiao Zhang[5], Liwei Chen[1,5,6], Chunhai Fan[1], Fangyuan Li[2,7,8*], Daishun Ling[1,3*]

1 Frontiers Science Center for Transformative Molecules, School of Chemistry and Chemical Engineering, School of Biomedical Engineering, National Center for Translational Medicine, State Key Laboratory of Oncogenes and Related Genes, Shanghai Jiao Tong University, Shanghai 200240, China

2 Institute of Pharmaceutics, College of Pharmaceutical Sciences, Zhejiang University, Hangzhou 310058, China

3 World Laureates Association (WLA) Laboratories, Shanghai 201210, China

4 Shanghai Synchrotron Radiation Facility, Shanghai Advanced Research Institute, Chinese Academy of Sciences, Shanghai 201204, China

5 In-situ Center for Physical Sciences, Shanghai Electrochemical Energy Device Research Center (SEED), Shanghai Jiao Tong University, Shanghai 200240, China

6 Future Battery Research Center, Global Institute of Future Technology, Shanghai Jiao Tong University, Shanghai 200240, China

7 Songjiang Research Institute, Songjiang Hospital, Shanghai Jiao Tong University School of Medicine, Shanghai, 201600, China

8 Key Laboratory of Precision Diagnosis and Treatment for Hepatobiliary and Pancreatic Tumor of Zhejiang Province, Hangzhou 310009, China

9 These authors contributed equally: Peihua Lin, Bo Zhang

Correspondence and requests for materials should be addressed to D.L. and F.L.

(*e-mail: dsling@sjtu.edu.cn; lfy@zju.edu.cn)

Supporting Information

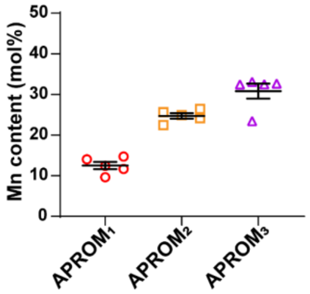

Supplementary Figure 1. The Mn content of $APROM_1$, $APROM_2$ and $APROM_3$ (n = 5 independent experiments). Data are presented as means ± s.e.m. Source data are provided as a Source Data file.

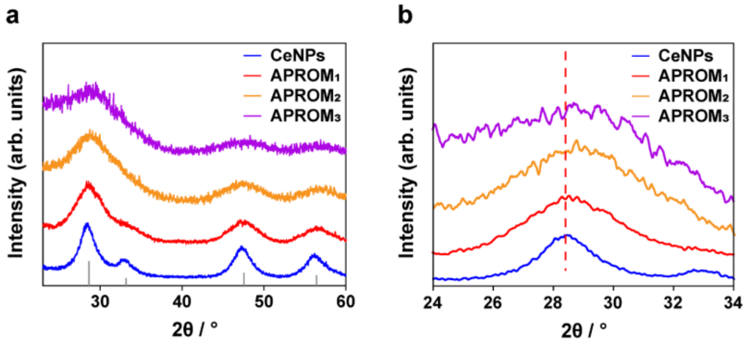

Supplementary Figure 2. a, X-ray diffraction (XRD) patterns of APROMs and CeNPs, indicating the cubic fluorite structure of APROMs and CeNPs (JCPDS card no. 34-0394). **b,** Enlarged XRD patterns around (111) plane of APROMs and CeNPs. Source data are provided as a Source Data file.

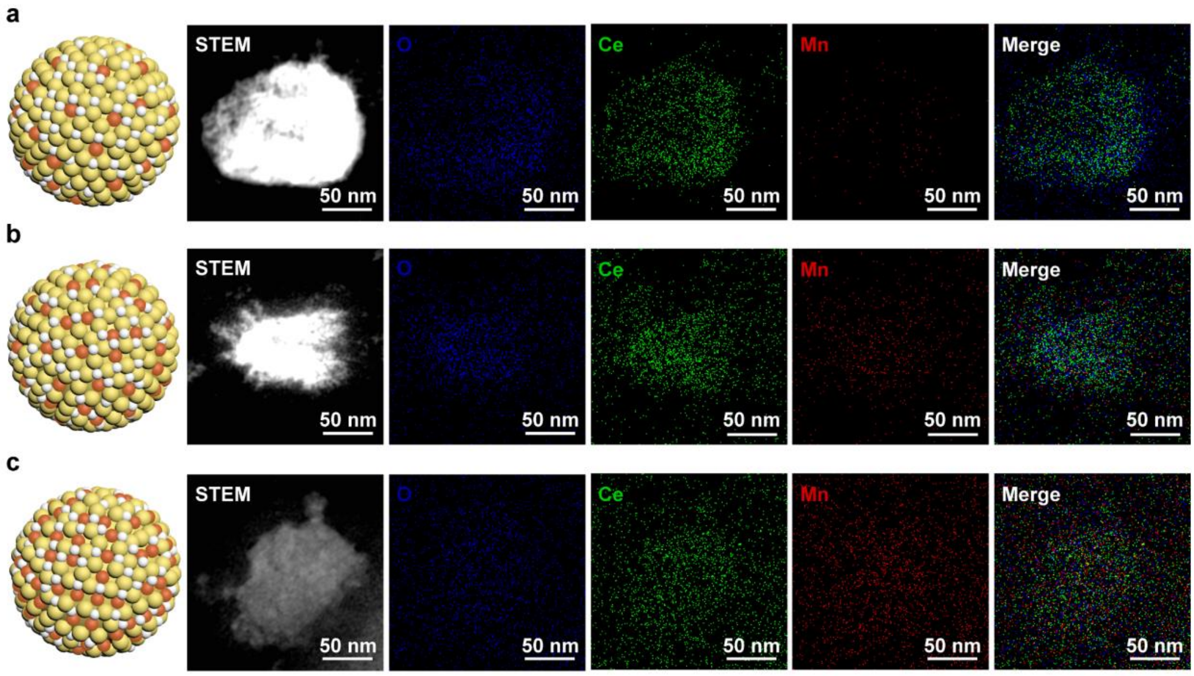

Supplementary Figure 3. Schematic illustration, scanning transmission electron microscopy (STEM) and corresponding energy dispersive spectroscopy (EDS) elemental mapping of O (blue), Ce (green), Mn (red) for $APROM_1$ (**a**), $APROM_2$ (**b**), and $APROM_3$ (**c**).

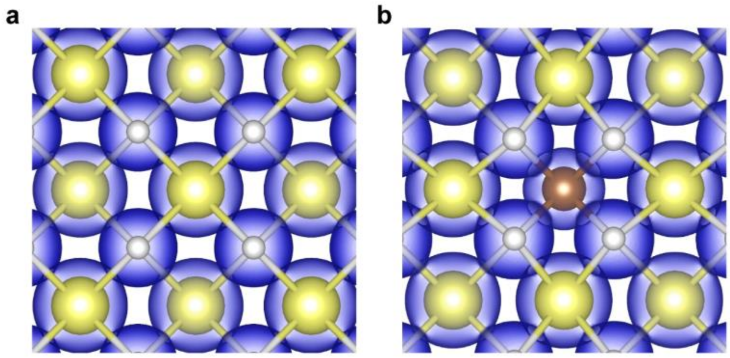

Supplementary Figure 4. Simulated charge-density isosurface plots of CeO_2 (**a**) and $Ce_{0.75}Mn_{0.25}O_2$ (**b**). The blue area represents a charge density of 0.05 e/bohr.

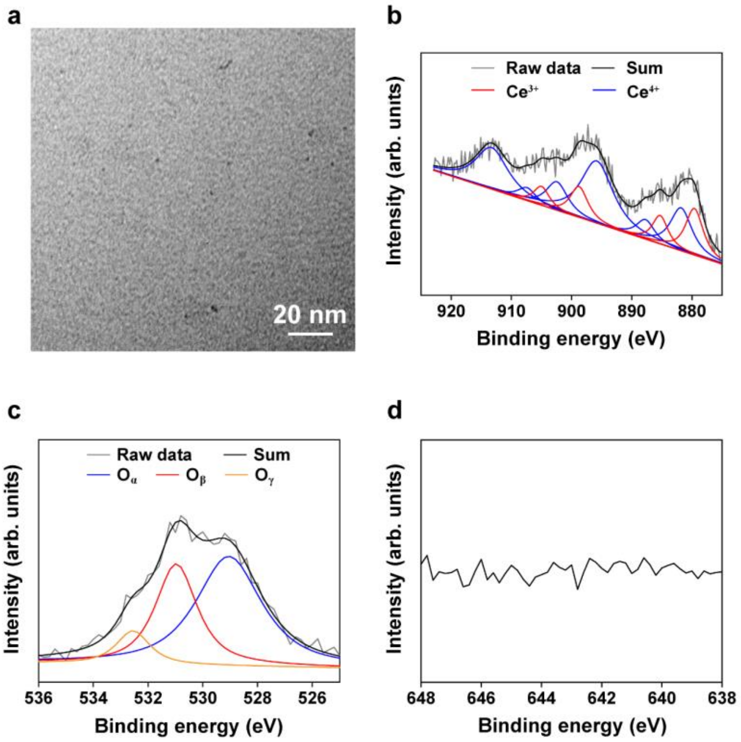

Supplementary Figure 5. a, TEM image of nanocrystals obtained after aging at 80°C for 0 h. **b-d,** High-resolution X-ray photoelectron spectroscopy (XPS) spectra of Ce 3*d* peaks (**b**), O1*s* peaks (**c**) and Mn 2*p* peaks (**d**) of nanocrystals obtain after aging for 0 h. Source data are provided as a Source Data file.

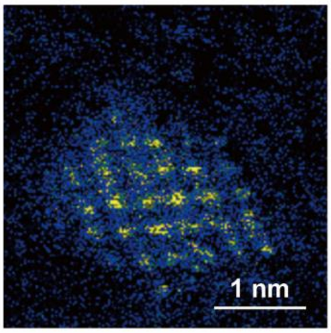

Supplementary Figure 6. The corresponding intensity map of the high-angle annular dark-field scanning transmission electron microscopy (HAADF-STEM) image of $APROM_2$ obtained after aging for 15 min.

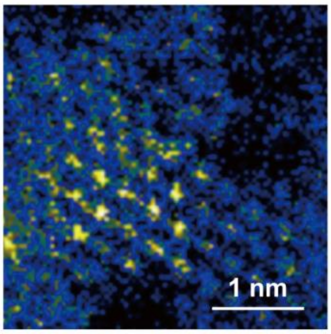

Supplementary Figure 7. The corresponding intensity map of the HAADF-STEM image of $APROM_2$ obtained after aging for 24 min.

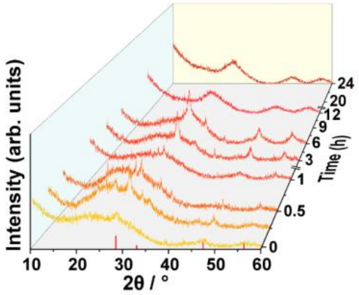

Supplementary Figure 8. XRD patterns of $APROM_2$ at different aging time. Source data are provided as a Source Data file.

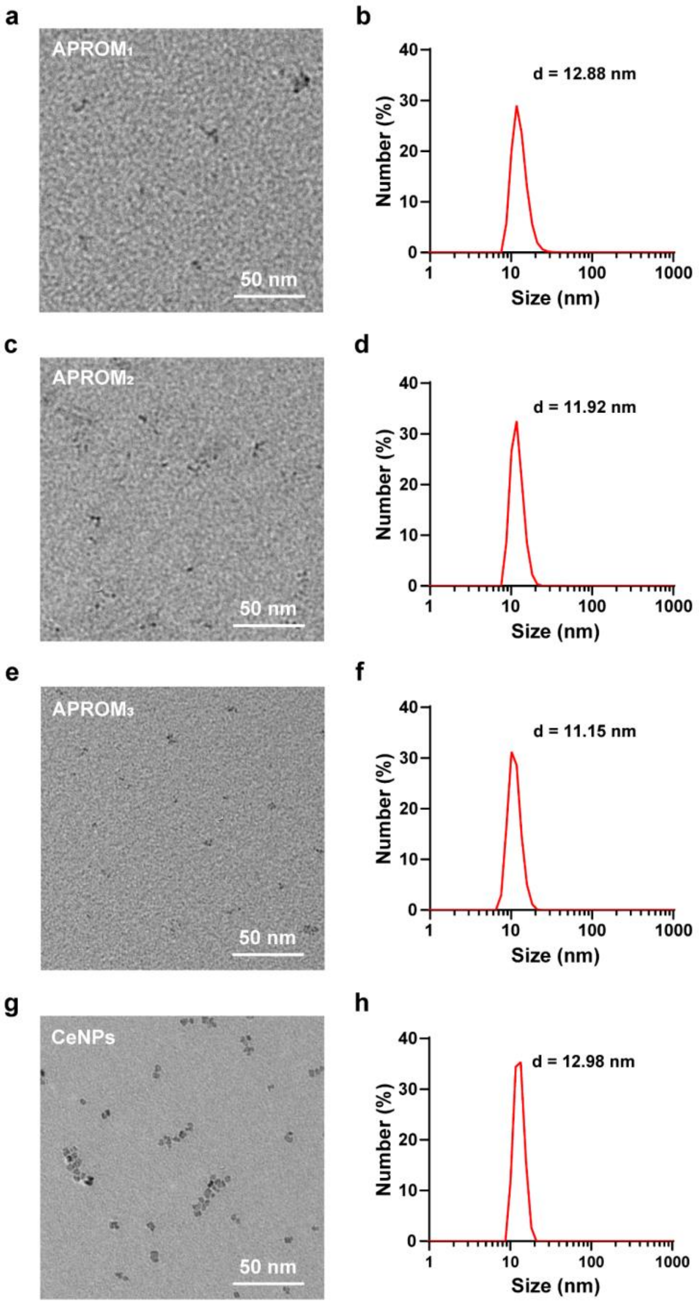

Supplementary Figure 9. TEM images and hydrodynamic diameter distribution of hydrophilic $APROM_1$ (**a, b**), $APROM_2$ (**c, d**), $APROM_3$ (**e, f**) and CeNPs (**g, h**), respectively. Source data are provided as a Source Data file.

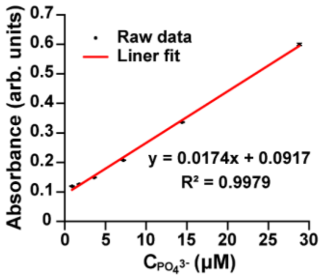

Supplementary Figure 10. Standard curve of phosphate (n = 3 independent experiments). Data are presented as means ± s.e.m. Source data are provided as a Source Data file.

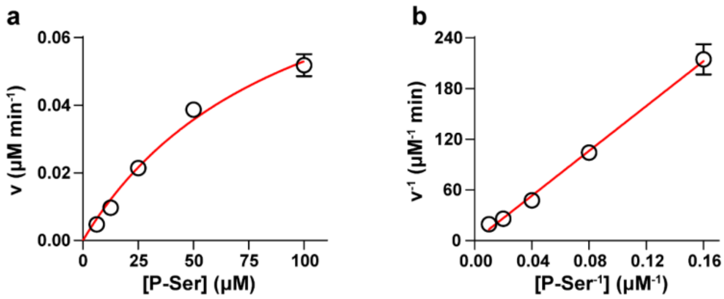

Supplementary Figure 11. Michaelis-Menten kinetics (**a**) and Lineweaver-Burk plotting (**b**) of APROM$_2$ obtained by adding different concentrations of P-Ser (n = 3 independent experiments). Data are presented as means ± s.e.m. Source data are provided as a Source Data file.

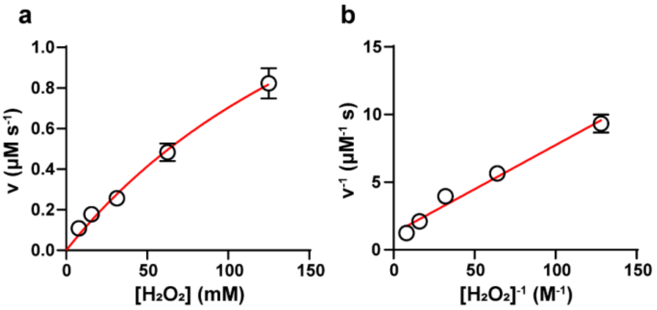

Supplementary Figure 12. Michaelis-Menten kinetics (**a**) and Lineweaver-Burk plotting (**b**) of CeNPs obtained by adding different concentrations of H_2O_2 (n = 3 independent experiments). Data are presented as means ± s.e.m. Source data are provided as a Source Data file.

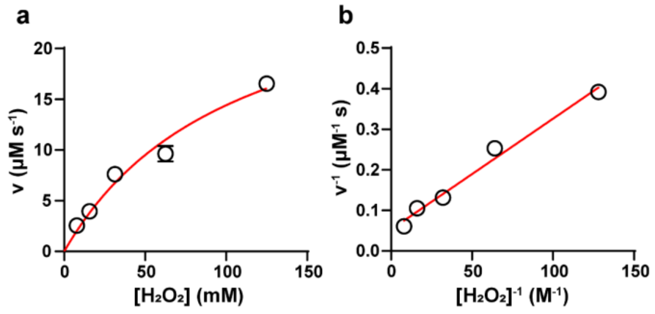

Supplementary Figure 13. Michaelis-Menten kinetics (**a**) and Lineweaver-Burk plotting (**b**) of natural catalase (CAT) obtained by adding different concentrations of H_2O_2 (n = 3 independent experiments). Data are presented as means ± s.e.m. Source data are provided as a Source Data file.

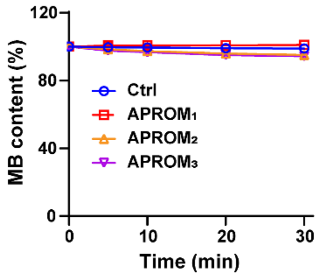

Supplementary Figure 14. UV-Vis absorbance of different methylene blue solution containing $APROM_1$, $APROM_2$, and $APROM_3$ (n = 3 independent experiments). No obvious degradation of MB is observed, indicating that APROMs would not generate hydroxyl radicals (·OH) via the Fenton-like reaction. Data are presented as means ± s.e.m. Source data are provided as a Source Data file.

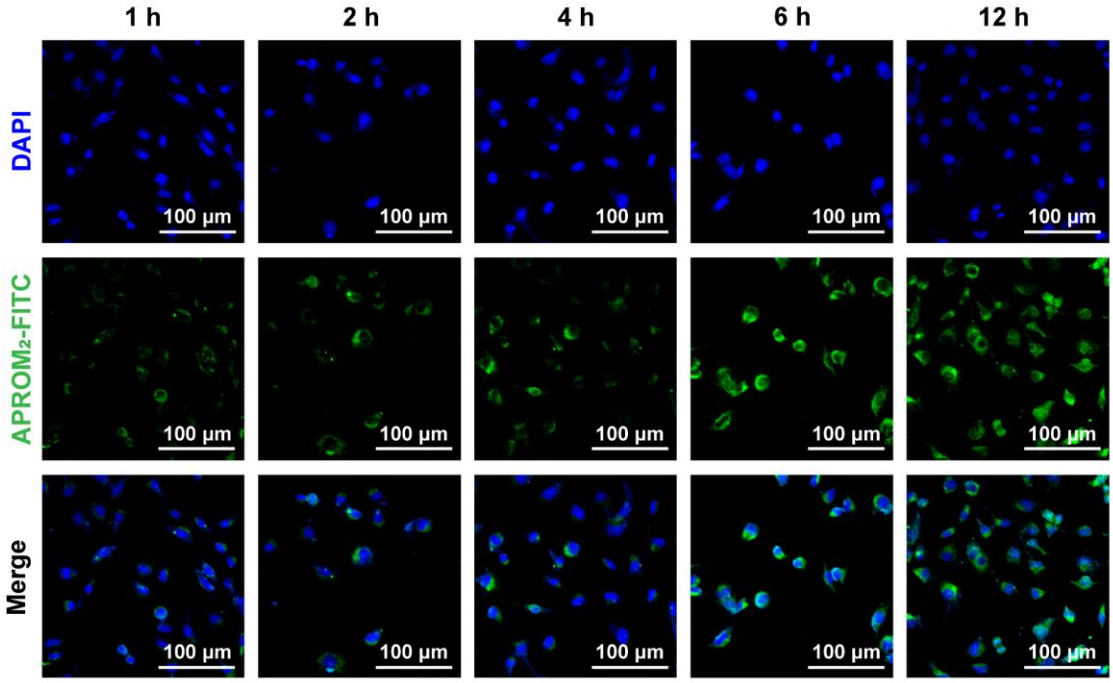

Supplementary Figure 15. Representative Confocal laser scanning microscopy (CLSM) images of SH-SY5Y cells incubated with APROM$_2$-FITC for various time points.

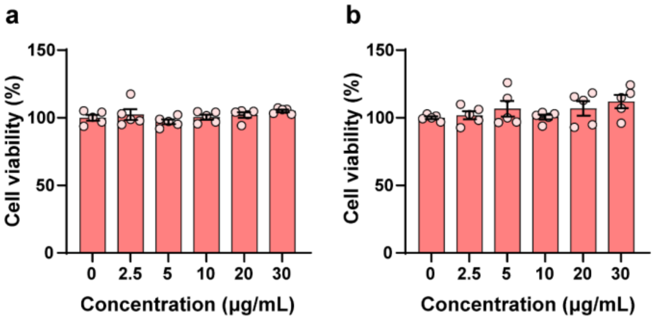

Supplementary Figure 16. Cell viability of SH-SY5Y cells after being treated with APROM$_2$ (**a**) or CeNPs (**b**) for 24 h (n = 5 biologically independent cultures). Data are presented as means ± s.e.m. Source data are provided as a Source Data file.

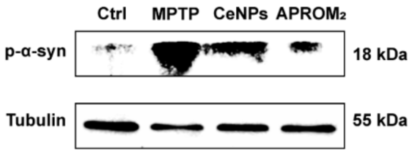

Supplementary Figure 17. Western blot analysis of p-α-syn in primary neurons with different treatments. Source data are provided as a Source Data file.

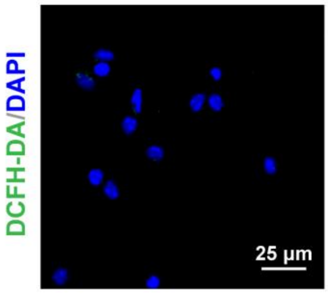

Supplementary Figure 18. Intracellular ROS levels in MPP^+ and NAC treated primary neurons. For comparison, the control and MPP^+ groups are presented in **Figure 5k**.

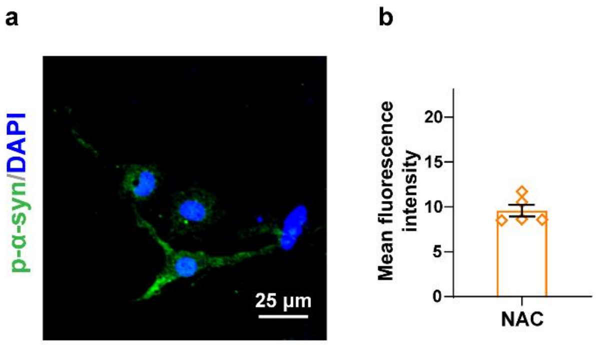

Supplementary Figure 19. Immunofluorescence (**a**) and mean fluorescence intensity (**b**) of p-α-syn in MPP^+ and NAC treated primary neurons (n = 5 biologically independent cultures). For comparison, the control and MPP^+ groups are presented in **Figure 5b**,**c**, and the mean fluorescence intensity of the control group is 1. Data are presented as means ± s.e.m. Source data are provided as a Source Data file.

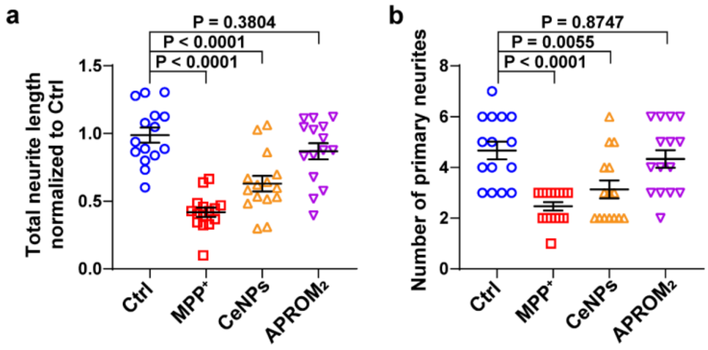

Supplementary Figure 20. Quantification of total neurite length (**a**) and number of primary neurites (**b**) in primary neurons after different treatments (n = 15 primary neurons). Data are presented as means ± s.e.m. Source data are provided as a Source Data file.

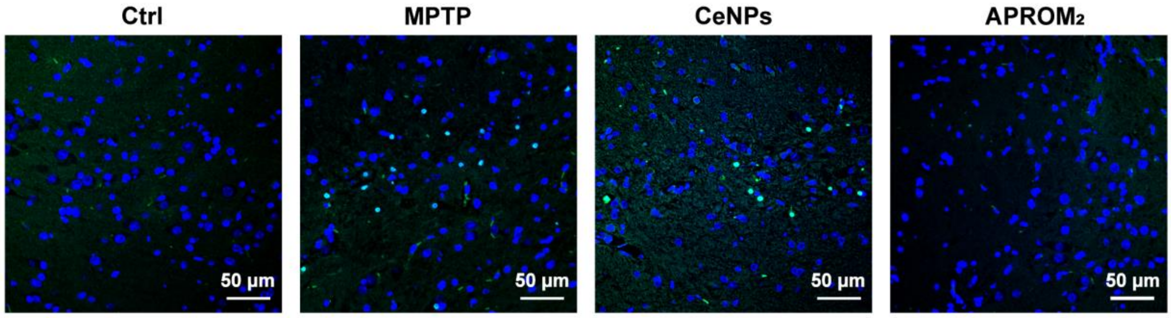

Supplementary Figure 21. Representative terminal deoxynucleotidyl transferase dUTP nick end labeling staining images in the SN.

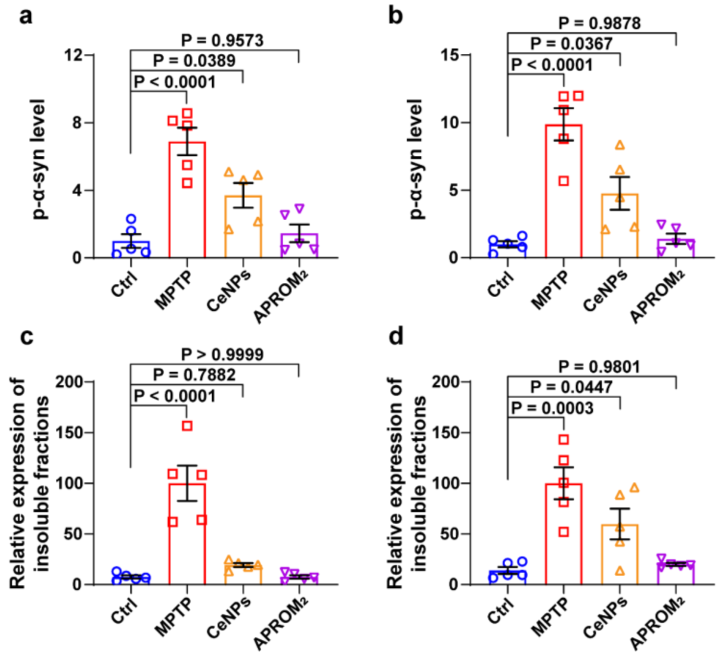

Supplementary Figure 22. The quantitative analysis of the p-α-syn level in the SN (**a**) and ST (**b**) after different treatments (n = 5 independent experiment). The insoluble fraction levels in the SN (**c**) and ST (**d**) after different treatments (n = 5 independent experiment). Data are presented as means ± s.e.m. Source data are provided as a Source Data file.

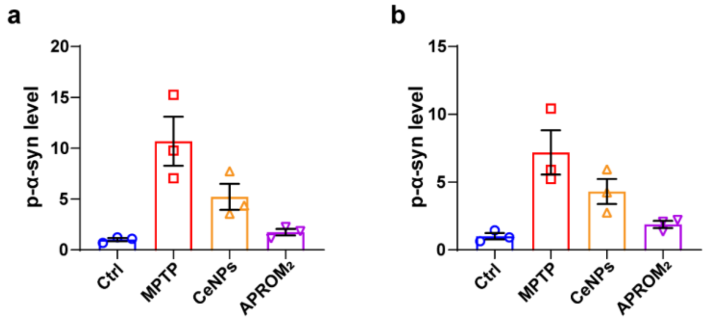

Supplementary Figure 23. The quantitative analysis of the p-α-syn level in the SN (**a**) and ST (**b**) after different treatments (n = 3 independent experiment). Data are presented as means ± s.e.m. Mice were treated with MPTP (20 mg/kg body weight) via subcutaneous injection four times at 2 h intervals to induce PD. The control group was treated with the equivalent volume of saline. 24 h later, mice were administrated with saline, CeNPs (2 mg/kg body weight), or $APROM_2$ (2 mg/kg body weight) by tail vein injection. 24 h after the last injection, mice were sacrificed for further quantitative analysis of the p-α-syn level in the SN and ST. Source data are provided as a Source Data file.

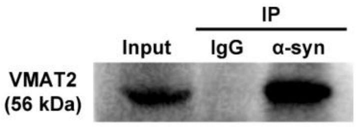

Supplementary Figure 24. Co-immunoprecipitation of α-syn with VMAT2 in the midbrain of $APROM_2$ treated PD mice. Source data are provided as a Source Data file.

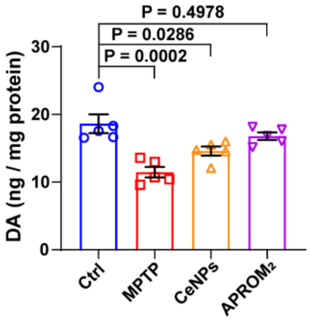

Supplementary Figure 25. The dopamine level in the SN of mice after different treatments (n = 5 biologically independent mice). Data are presented as means ± s.e.m. Source data are provided as a Source Data file.

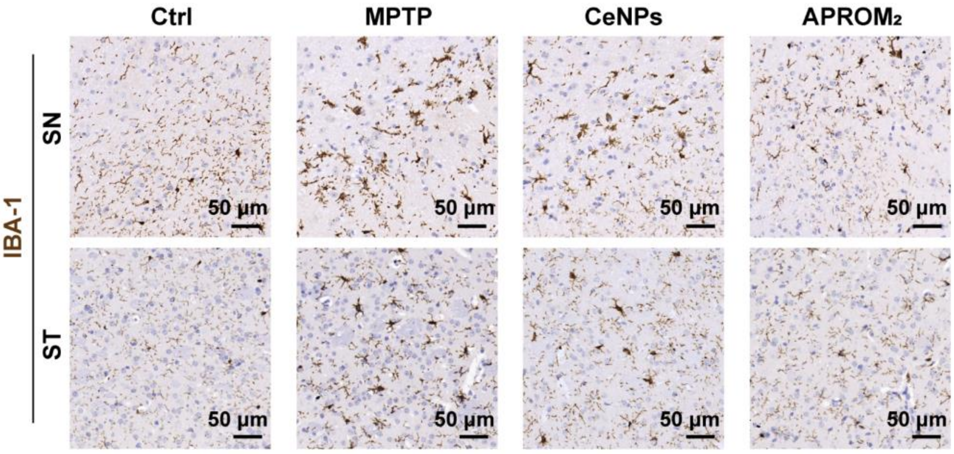

Supplementary Figure 26. Representative immunofluorescence staining images of 4-HNE.

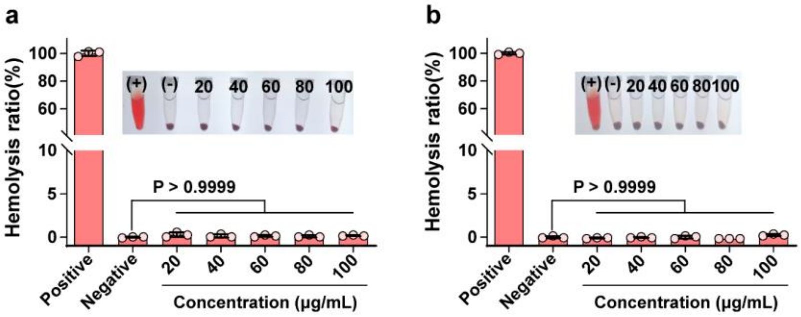

Supplementary Figure 27. Quantitative results and photographs of hemolysis activity of CeNPs (**a**) and APROM$_2$ (**b**) with different concentrations (n = 3 independent experiment). Data are presented as means ± s.e.m. Source data are provided as a Source Data file.

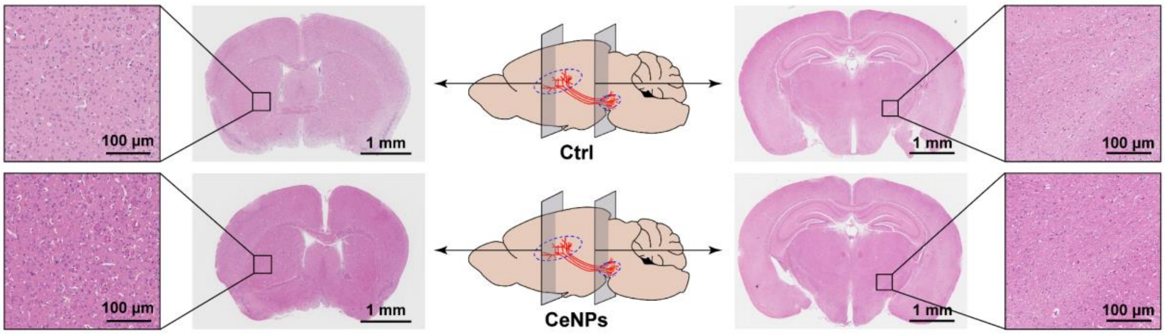

Supplementary Figure 28. Schematic of the midbrain section and histological assay with H&E staining for the SN and ST of mice after different treatments.

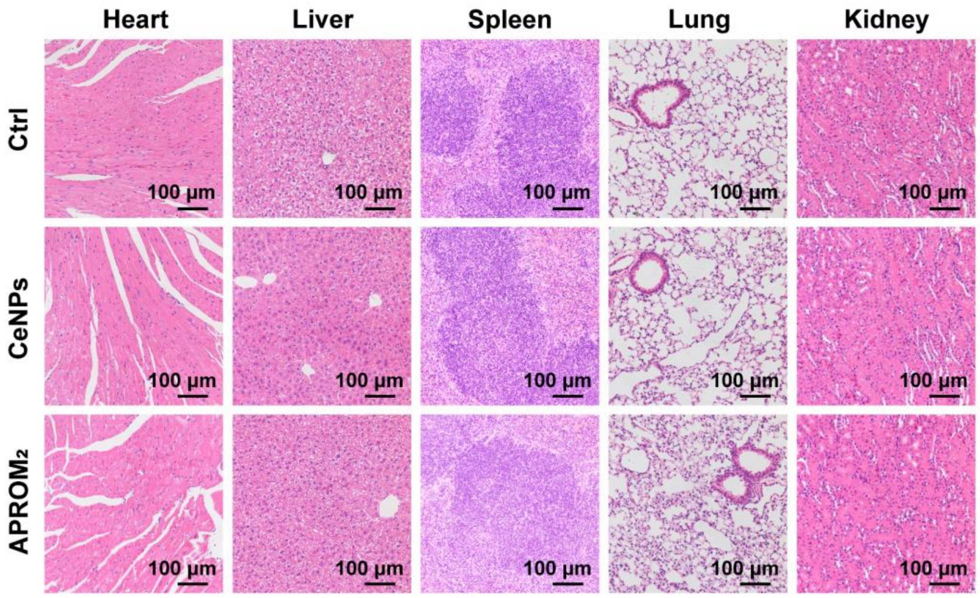

Supplementary Figure 29. Histological assay with H&E staining for major organ tissues of saline, CeNPs or $APROM_2$ treated mice.

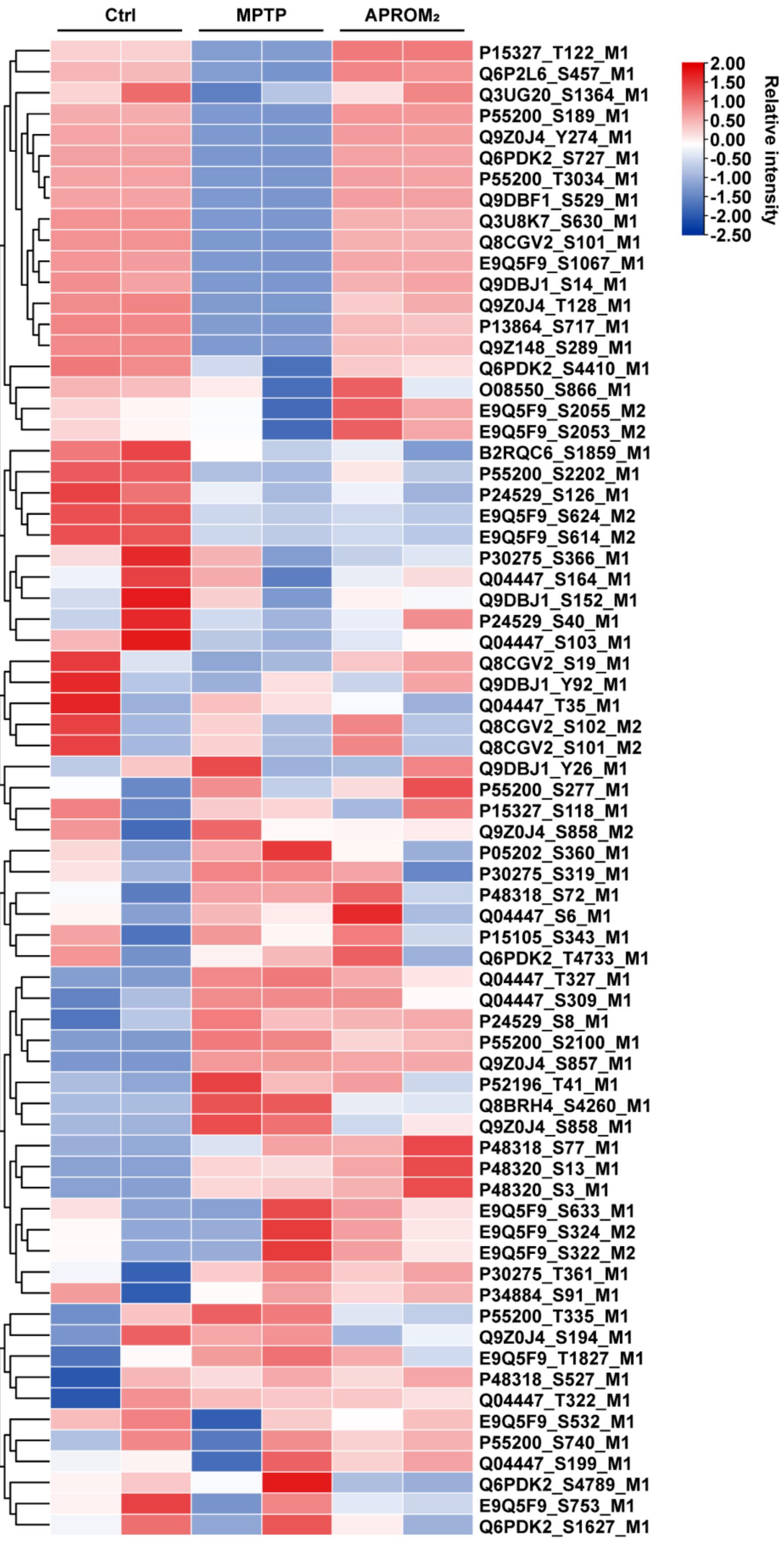

Supplementary Figure 30. The heatmap of amino acid metabolism related phospho-proteins. Source data are provided as a Source Data file.

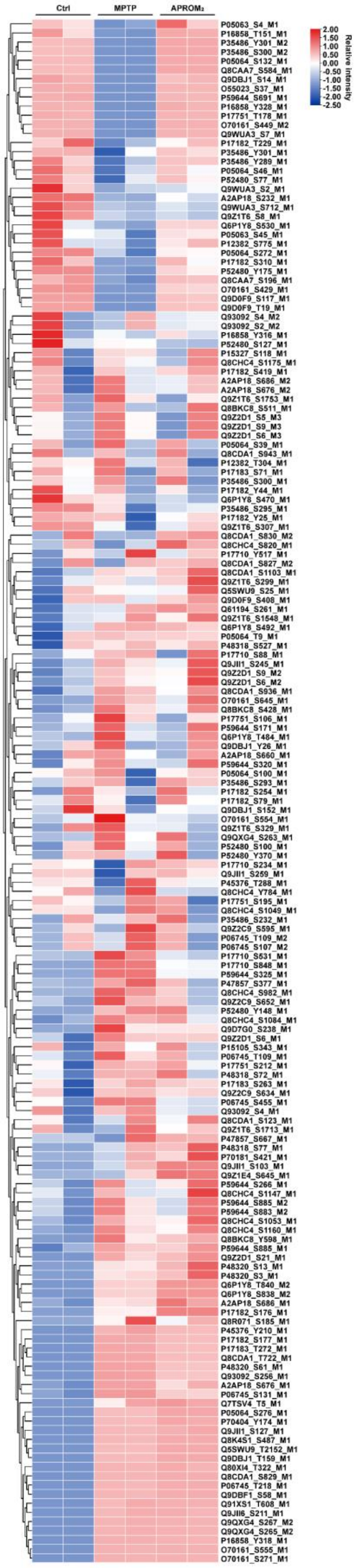

Supplementary Figure 31. The heatmap of carbohydrate metabolism related phospho-proteins. Source data are provided as a Source Data file.

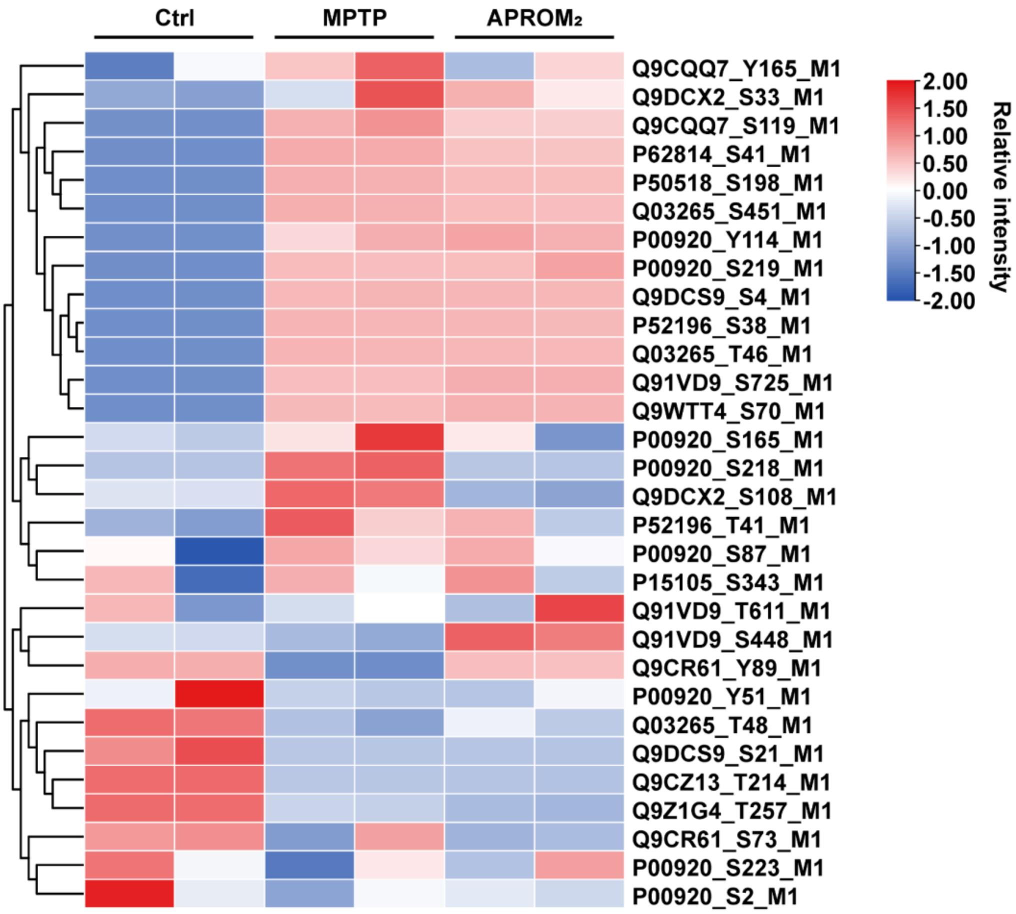

Supplementary Figure 32. The heatmap of energy metabolism related phospho-proteins. Source data are provided as a Source Data file.

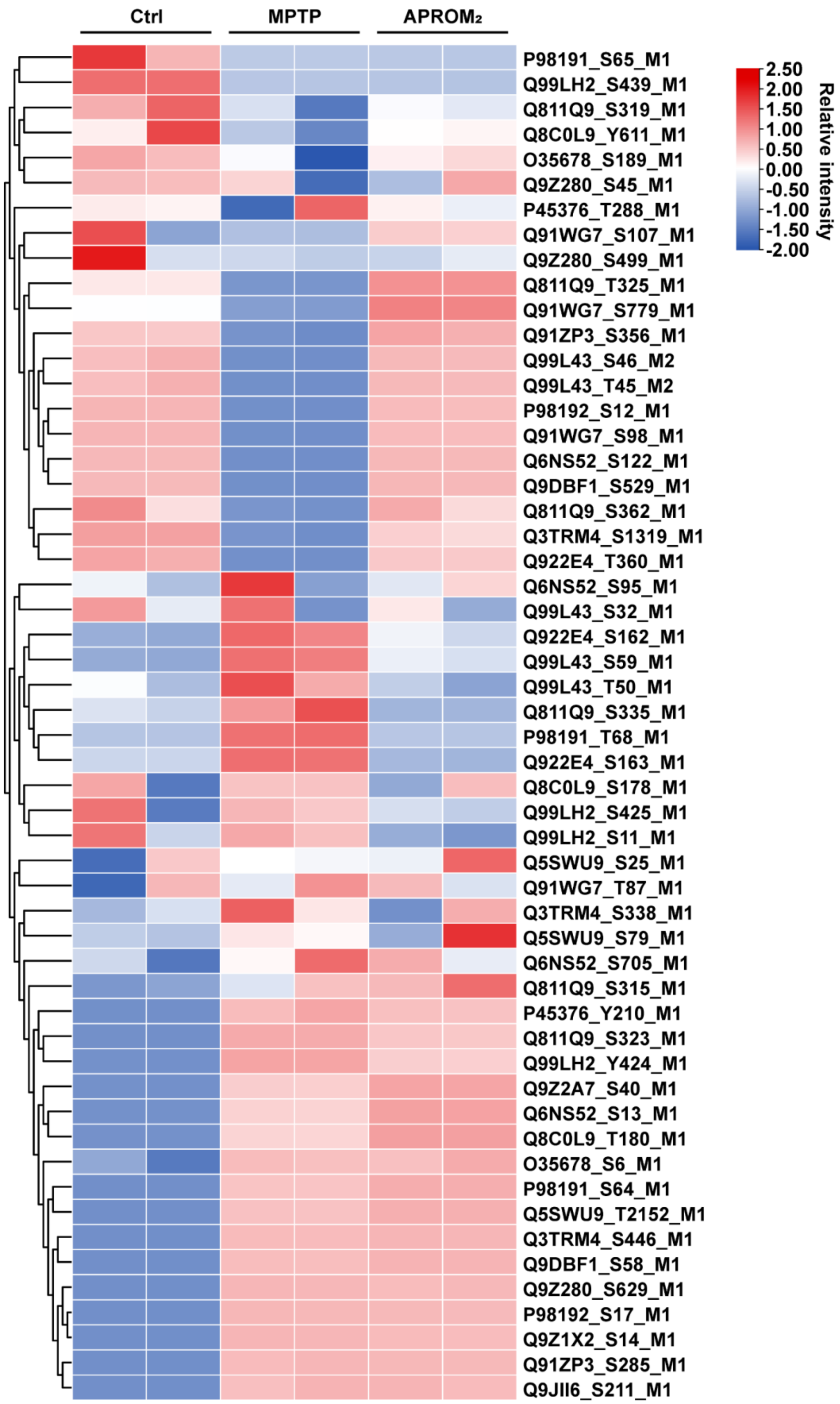

Supplementary Figure 33. The heatmap of lipid metabolism related phospho-proteins. Source data are provided as a Source Data file.

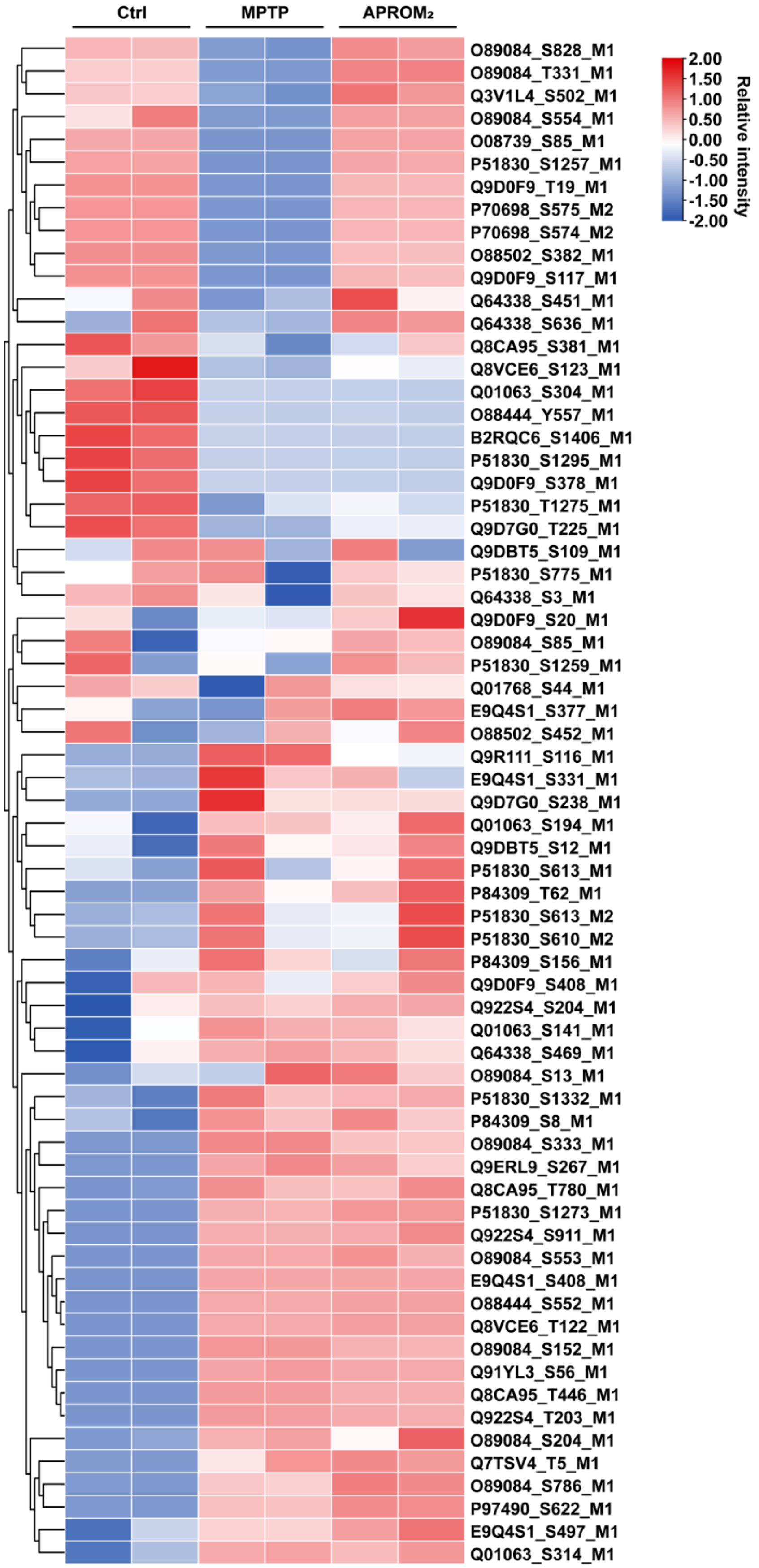

Supplementary Figure 34. The heatmap of nucleotide metabolism related phospho-proteins. Source data are provided as a Source Data file.

Supplementary Table 1. Properties of the as-synthesized APROMs and CeNPs.

Samples	Ce ions (%)	
	Ce^{3+}	Ce^{4+}
CeNPs	23.21	76.79
$APROM_1$	28.35	71.65
$APROM_2$	39.72	60.28
$APROM_3$	31.16	68.84

Supplementary Table 2. The reaction energy barriers of self-aggravating surface oxygen vacancy-driven cation exchange reaction on the model of CeO_2 and CeO_{2-x} model, including the movement energy (ΔE_{mo}), and migration energy (ΔE_{mi}).

Model	Cation	ΔE_{mo} (eV)	ΔE_{mi} (eV)
CeO_2	Ce	10.27	14.91
	Mn	1.83	6.09
CeO_{2-x}	Ce	9.02	12.99
	Mn	0.62	4.75

Supplementary Table 3. Kinetics parameters of $APROM_2$ for protein phosphatase-mimetic activity.

Catalyst	Substrate	K_m (mM)	V_{max} (μM s^{-1})	k_{cat} (s^{-1})
$APROM_2$	P-Ser	39.17	0.49	2.47×10^2
$APROM_2$	p-α-syn	0.175	1.5×10^{-3}	0.764

K_m is the Michaelis-Menten constant, V_{max} is the maximal reaction velocity, and k_{cat} is the catalytic constant.

Supplementary Table 4. Kinetics parameters of $APROM_2$ and natural SOD with xanthine as the substrate for SOD-mimetic activity.

Catalyst	K_m (mM)	V_{max} (μM s^{-1})	k_{cat} (s^{-1})
$APROM_2$	50.70	32.46	1.63×10^5
Natural SOD	7.63	4.20	1.57×10^2

K_m is the Michaelis-Menten constant, V_{max} is the maximal reaction velocity, and k_{cat} is the catalytic constant.

Supplementary Table 5. Kinetics parameters of natural CAT, CeNPs, and $APROM_2$ with H_2O_2 as the substrate for CAT-mimetic activity.

Catalyst	K_m (mM)	V_{max} (μM s^{-1})	k_{cat} (s^{-1})
Natural CAT	51.57	18.88	7.90×10^4
CeNPs	53.53	0.82	1.81×10^3
$APROM_2$	15.42	3.87	3.88×10^3

K_m is the Michaelis-Menten constant, V_{max} is the maximal reaction velocity, and k_{cat} is the catalytic constant.
