# Supplementary material for: An artificial protein modulator reprogramming neuronal protein functions
Source: Nat Commun. 2024 Mar 6;15:2039. doi: 10.1038/s41467-024-46308-6 (PMC10917760; doi:10.1038/s41467-024-46308-6)
Supplement: Supplementary file 3 — Reporting Summary [file 41467_2024_46308_MOESM3_ESM.pdf]

nature portfolio

Corresponding author(s): Daishun Ling, Fangyuan Li

Last updated by author(s): Jan 26, 2024

Reporting Summary

Nature Portfolio wishes to improve the reproducibility of the work that we publish. This form provides structure for consistency and transparency in reporting. For further information on Nature Portfolio policies, see our Editorial Policies and the Editorial Policy Checklist.

Statistics

For all statistical analyses, confirm that the following items are present in the figure legend, table legend, main text, or Methods section.

n/a	Confirmed	
☐	☒	The exact sample size (*n*) for each experimental group/condition, given as a discrete number and unit of measurement
☐	☒	A statement on whether measurements were taken from distinct samples or whether the same sample was measured repeatedly
☐	☒	The statistical test(s) used AND whether they are one- or two-sided<br>*Only common tests should be described solely by name; describe more complex techniques in the Methods section.*
☒	☐	A description of all covariates tested
☒	☐	A description of any assumptions or corrections, such as tests of normality and adjustment for multiple comparisons
☐	☒	A full description of the statistical parameters including central tendency (e.g. means) or other basic estimates (e.g. regression coefficient) AND variation (e.g. standard deviation) or associated estimates of uncertainty (e.g. confidence intervals)
☐	☒	For null hypothesis testing, the test statistic (e.g. *F*, *t*, *r*) with confidence intervals, effect sizes, degrees of freedom and *P* value noted<br>*Give P values as exact values whenever suitable.*
☒	☐	For Bayesian analysis, information on the choice of priors and Markov chain Monte Carlo settings
☒	☐	For hierarchical and complex designs, identification of the appropriate level for tests and full reporting of outcomes
☐	☒	Estimates of effect sizes (e.g. Cohen's *d*, Pearson's *r*), indicating how they were calculated

Our web collection on statistics for biologists contains articles on many of the points above.

Software and code

Policy information about availability of computer code

Data collection	Characterization of nanomaterials properties were carried out from transmission electron microscopy (Hitachi HT7700, Japan), high-resolution transmission electron microscopy (FEI Tecnai F20, USA), high-angle annular dark-field scanning transmission electron microscopy (Titan ChemiSTEM, USA), XPS (Thermo Scientific ESCALAB 250 Xi, UK), XRD (Rigaku D/Max-2550 PC, Japan), in situ XRD (Bruker D8 Advanced, Germany), Raman spectrometer (LabRAM HR evolution, France) and ToF-SIMS (ION-TOF, Germany). The absorbance was measured by using a UV-Vis spectrophotometer UV-2600 (Shimadzu, Japan) or a microplate reader (Bio Tech, USA). Confocal images were captured by confocal laser scanning microscopy (Olympus FV1200, Japan). Oxygen generation was measured by an oxygen electrode on Dissolved Oxygen Meter JPSJ-606L (Leici, China).
Data analysis	Quantitative analysis of the fluorescence images was performed using Fiji (version 1.54f). Statistical comparisons were analyzed using Origin 2018 (version b9.5.0.193) and Graphpad Prism (version 8.0). The density functional theory calculations were carried out using Quantum espresso (version 6.7).

For manuscripts utilizing custom algorithms or software that are central to the research but not yet described in published literature, software must be made available to editors and reviewers. We strongly encourage code deposition in a community repository (e.g. GitHub). See the Nature Portfolio guidelines for submitting code & software for further information.

Data

Policy information about availability of data

All manuscripts must include a data availability statement. This statement should provide the following information, where applicable:

- Accession codes, unique identifiers, or web links for publicly available datasets
- A description of any restrictions on data availability
- For clinical datasets or third party data, please ensure that the statement adheres to our policy

The authors declare that all data generated in this study are provided in the Figures, Supplementary information, and Source Data file. Source data are provided with this paper.

Research involving human participants, their data, or biological material

Policy information about studies with human participants or human data. See also policy information about sex, gender (identity/presentation), and sexual orientation and race, ethnicity and racism.

Reporting on sex and gender	N/A
Reporting on race, ethnicity, or other socially relevant groupings	N/A
Population characteristics	N/A
Recruitment	N/A
Ethics oversight	N/A

Note that full information on the approval of the study protocol must also be provided in the manuscript.

Field-specific reporting

Please select the one below that is the best fit for your research. If you are not sure, read the appropriate sections before making your selection.

☒ Life sciences ☐ Behavioural & social sciences ☐ Ecological, evolutionary & environmental sciences

For a reference copy of the document with all sections, see nature.com/documents/nr-reporting-summary-flat.pdf

Life sciences study design

All studies must disclose on these points even when the disclosure is negative.

Sample size	Sample size choice was based on previous studies (ref. Jiang, W., Li, Q., Zhang, R. et al, 2023. https://doi.org/10.1038/s41467-023-43870-3; Kim, T., Kim, H. J., Choi, W. et al, 2022. https://doi.org/10.1038/s41551-022-00965-4; Kim, D., Yoo, J. M., Hwang, H. et al, 2018. https://doi.org/10.1038/s41565-018-0179-y), not predetermined by a statistical method. Sample sizes were indicated in the legends of each Figure and Supplementary Figure.
Data exclusions	No data were excluded.
Replication	We confirm all attempts at replication were successful. Replicates were conducted for all experiments quantified as described in the Figure legends.
Randomization	All samples were randomly allocated into experimental groups.
Blinding	Investigators were not blinded for nanomaterial synthesis, due to any prior knowledge does not affect synthesis in this study. For nanomaterial's characterization, cell experiments and in vivo experiments, the investigators were blinded to group allocation during data collection and analysis.

Reporting for specific materials, systems and methods

We require information from authors about some types of materials, experimental systems and methods used in many studies. Here, indicate whether each material, system or method listed is relevant to your study. If you are not sure if a list item applies to your research, read the appropriate section before selecting a response.

Materials & experimental systems

n/a	Involved in the study
☐	☒ Antibodies
☐	☒ Eukaryotic cell lines
☒	☐ Palaeontology and archaeology
☐	☒ Animals and other organisms
☒	☐ Clinical data
☒	☐ Dual use research of concern
☒	☐ Plants

Methods

n/a	Involved in the study
☒	☐ ChIP-seq
☒	☐ Flow cytometry
☒	☐ MRI-based neuroimaging

Antibodies

Antibodies used

anti-phospho-synuclein alpha (Ser129) (cat. no. AF3285, Clone name: Rabbit polyclonal antibody, Affinity Biosciences), anti-VMAT2 (cat. no. PA5-112713, Clone name: Rabbit polyclonal antibody, Thermo Fisher Scientific), anti-VMAT2 (cat. no. ab259970, Clone name: Rabbit monoclonal antibody, Abcam), anti-VAMP2 (cat. no. DF6381, Clone name: Rabbit polyclonal antibody, Affinity Biosciences), anti-alpha Synuclein (cat. no. OM239190, Clone name: Mouse monoclonal antibody, Omnimabs), anti-alpha Synuclein (cat. no. FNab09891, Clone name: Mouse monoclonal antibody, FineTest), anti-TH (cat. no. 25859-1-AP, Clone name: Rabbit polyclonal antibody, Proteintech), anti-IBA1 (cat. no. BM5765, Clone name: Rabbit monoclonal antibody, Boster), anti-4-HNE (cat. no. bs-6313R, Clone name: Rabbit polyclonal antibody, Bioss), FITC Conjugated AffiniPure Goat Anti-rabbit IgG (H+L) (cat. no. BA1105, Boster), TRITC Conjugated AffiniPure Goat Anti-rabbit IgG (H+L) (cat. no. BA1090, Boster), FITC Conjugated AffiniPure Goat Anti-mouse IgG (H+L) (cat. no. BA1101, Boster), Rabbit IgG control Polyclonal antibody (cat. no. 30000-0-AP, Proteintech), HRP-conjugated Affinipure Goat Anti-Rabbit IgG(H+L) (cat. no. SA00001-2, Proteintech), HRP-conjugated Affinipure Goat Anti-Mouse IgG(H+L) (cat. no. SA00001-1, Proteintech)

Validation

anti-phospho-synuclein alpha (Ser129) antibody
https://www.affbiotech.cn/goods-1452-AF3285-Phospho_Synuclein_alpha_Ser129_Antibody.html

anti-VMAT2 antibody
https://www.thermofisher.cn/cn/zh/antibody/product/VMAT2-Antibody-Polyclonal/PA5-112713

anti-VMAT2 antibody
https://www.abcam.cn/products/primary-antibodies/vmat2-antibody-epr24197-51-ab259970.html?productWallTab=ShowAll#lb

anti-VAMP2 antibody
https://www.affbiotech.cn/goods-5183-DF6381-VAMP2_Antibody.html

anti-alpha Synuclein antibody
http://www.omnimabs.com/antibody_alpha_Synuclein_antibody-OM239190.html

anti-alpha Synuclein antibody
https://www.fn-test.cn/product/fnab09891/

anti-TH antibody
https://www.ptgcn.com/products/TH-Antibody-25859-1-AP.htm

anti-IBA1 antibody
https://www.boster.com.cn/index/products/productsDetail?goods_sn=BM5765

anti-4-HNE antibody
http://www.bioss.com.cn/prolook_03.asp?id=AF08169606010648&pro37=1

FITC Conjugated AffiniPure Goat Anti-rabbit IgG (H+L)
https://www.boster.com.cn/index/products/productsDetail?goods_sn=BA1105

TRITC Conjugated AffiniPure Goat Anti-rabbit IgG (H+L)
https://www.boster.com.cn/index/products/productsDetail?goods_sn=BA1090

FITC Conjugated AffiniPure Goat Anti-mouse IgG (H+L)
https://www.boster.com.cn/index/products/productsDetail?goods_sn=BA1101

Rabbit IgG control Polyclonal antibody
https://www.ptglab.com/products/IgG-control-Antibody-30000-0-AP.htm

HRP-conjugated Affinipure Goat Anti-Rabbit IgG(H+L)
https://www.ptglab.com/products/HRP-conjugated-Affinipure-Goat-Anti-Rabbit-IgG-H-L-secondary-antibody.htm

HRP-conjugated Affinipure Goat Anti-Mouse IgG(H+L)
https://www.ptglab.com/products/HRP-conjugated-Affinipure-Goat-Anti-Mouse-IgG-H-L-secondary-antibody.htm

Eukaryotic cell lines

Policy information about cell lines and Sex and Gender in Research

Cell line source(s)	SH-SY5Y cells (CL-0208) were obtained from Procell Life Science&Technology Co.,Ltd (Wuhan, China).
Authentication	Cells were identified by Short Tandem Repeat (STR) method.
Mycoplasma contamination	The cell line was tested negative for mycoplasm contamination per suppliers.
Commonly misidentified lines (See ICLAC register)	No commonly misidentified lines were used.

Animals and other research organisms

Policy information about studies involving animals; ARRIVE guidelines recommended for reporting animal research, and Sex and Gender in Research

Laboratory animals	Primary neurons were prepared from C57BL/6 mice within 24 h of birth (male, Shanghai SLAC Laboratory Animal Co. Ltd.) C57BL/6 mice (male, 7-8 weeks, Shanghai SLAC Laboratory Animal Co. Ltd.) were used in in vivo experiments for the assessment of PD treatment. All mice were housed in a specific pathogen-free environment at 21±1°C and 60±5% humidity, with a 12h light-dark cycle.
Wild animals	Wild animals were not involved in this study.
Reporting on sex	Male mice were used in the studies of in vitro and in vivo experiments, and there was no other sex bias in the animals used.
Field-collected samples	Field-collected samples were not involved in this study.
Ethics oversight	The use and care of the mice were in accordance with the guidelines of the Institutional Animal Care and Use Committee of Zhejiang University and Shanghai Jiao Tong University. All procedures were approved by the Institutional Animal Care and Use Committee of Zhejiang University and Shanghai Jiao Tong University.

Note that full information on the approval of the study protocol must also be provided in the manuscript.

Plants

Seed stocks	N/A
Novel plant genotypes	N/A
Authentication	N/A
